# General Principles of Neurorobotic Models Employing Entrainment and Chaos Control

**DOI:** 10.3389/fnbot.2019.00032

**Published:** 2019-05-29

**Authors:** Kole Harvey

**Affiliations:** Independent Researcher, Dublin, Ireland

**Keywords:** chaos control, entrainment, embodied cognition, CPGs, coordination dynamics

## Abstract

Neurorobotic models are perfect candidates for proving the validity of embodied/dynamical approaches to cognition. In order to control bodies of arbitrary complexity in complex environments, it is necessary to coordinate vast numbers of sensory and motor components. In this review, we took at several studies of neurorobotics and generalize common principles of how they achieve this massive feat, relying on the key concepts of entrainment and chaos control. We discuss current limitations and ways that these techniques could be expanded to cover more wide ranges of behavior, for example by taking inspiration from ecological psychology.

## Introduction

Neurorobotic models are perfect candidates for proving the validity of embodied/dynamical approaches to cognition (Hoffmann and Pfeifer, 2018). One of these approaches is radical embodied cognition (REC), which treats the brain, body, and environment in terms of coupled dynamical systems and ecological psychology (Chemero, 2011). Here the mind is not isolated from the world, but distributed across brain, body, and environment; perception of the world is direct and does not need to be inferred or synthesized through symbolic representations.

We believe there is a pressing need to expand the scope of these models, which so far have been restricted to demonstrations of “minimally cognitive behavior” (Brooks, 1991; Barandiaran and Moreno, 2006; van Duijn et al., 2006). Advancements made in the space of embodied agents would be hugely fruitful to the study of both artificial intelligence and cognitive science, as any behaviors successfully revealed will be inherently adaptive to real-world conditions and thus will not suffer to the limited application to “toy worlds” or “test settings.”

According to the REC view, complex cognitive behaviors would require a body with sufficient complexity to support them. However, as the field of robotics has no doubt learned the hard way, it becomes a challenging problem to solve the coordination problem between the hundreds to thousands of sensors and motors on such a body. In fact, this could be the very problem evolution was trying to solve by allowing cognitively sophisticated organisms to emerge in the first place (Barrett, 2018). The state space of any coupled brain-body-environment system is impossibly large, and no doubt contains an almost infinite number of aperiodic, unpredictable attractors (Cvitanovic et al., 2016). A key issue to solve is how to navigate this space in order to find solutions corresponding to useful behaviors that can be enacted by a robotic agent.

## Embodied Behavior and the Control of Chaos

Despite the vast magnitude of the brain-body-environment state space, we will see that models inspired by the dynamics of real organisms are able to traverse it effectively, by relying on the properties of chaotic systems to find patterns of coordination in a tractable manner. For example, Crutchfield has discussed how chaos could be used as a source of generativity (Crutchfield, 2012), and this is further discussed in the context of neural activity in Faure and Korn (2001), which leads us to imagine how a large behavioral repertoire could be developed from simple neural resources.

The studies we refer to in this letter thus all share a common objective: to search out those useful attractors in the state space of the brain-body-environment system which correspond to adaptive behaviors and to stabilize them into usable solutions.

There have been several approaches for controlling chaos given in the literature, all of which capture and stabilize unstable periodic orbits (UPOs) in the chaotic attractor landscape. One way is through the use of external perturbation to stabilize UPOs, known as “Noise Induced Order” 一郎津田 and 健司松本 (1985).

Another solution by Steingrube et al. (2010) is to let the system simultaneously detect and stabilize periodic orbits using time-delayed feedback and relies on the techniques of chaos control outlined in Ott et al. (1990) and Pikovsky et al. (2002). In this model, a single central pattern generator (CPG) is used to generate multiple complex patterns. The CPG signal is chaotic until control is applied, and so the control signal can be thought of effectively taming chaos and giving the model control over a large number of different periodic orbits, each of which can then be exploited by the robot it controls. For example, each of these orbits can be mapped to different behavioral gaits such as walking, running, or trotting. This efficient kind of “mode selection” relies on very little neural resources, and allows for simple learning of sensorimotor maps.

This is extended further by Ren et al. (2015), whereby multiple CPGs are used together and can act either in a synchronized fashion similar to Steingrube's single circuit or in a desynchronized fashion which allows for recovery of stable behavior even after limbs of the robot are disabled.

In Pitti et al. (2005), small, well-timed feedback actions are used to either stabilize chaotic activity into periodic orbits, thus entraining the system to a behavior, or to destabilize it, and induce a transition to a new behavior.

Another method by Shim and Husbands (2012), uses a bifurcation parameter to adaptively change the level of chaos in the system dependant on how well the current behavior is performing (based on a feedback signal which measures performance), allowing the system to escape from low performing behaviors.

Entrainment of the brain-body-environment system into periodic orbits, and in particular the control of chaos to search for and stabilize those orbits, offers a promising direction for the design of neural circuits that allow an embodied system to traverse complex motor spaces and find novel solutions which are adaptive to the behavioral situation. Importantly, such circuits can allow embodied cognitive systems to generate a variety of different behaviors in bodies with many degrees of freedom while using minimal neural hardware to do so. Next, we will discuss certain aspects of such a system in practice, such as the entrainment of the brain and body to the environment (**Principle 1**), the mutual cooperation between neural components (**Principle 2**), the flexible and rapid responses afforded to chaotic systems through itinerancy (**Principle 3**), the storage of coherent behavioral patterns through neural plasticity (**Principle 4**), and the dependence of the system on environmental information as viewed from an ecological perspective (**Principle 5**). We shall then conclude by considering remaining issues for the field in light of the outlined principles.

### Principle 1: Entrainment

In opposition to the traditional view of motor control as the design and execution of “motor plans,” a self-organized approach which describes embodied behavior in terms of emergent dynamic interactions between the central nervous system and the musculoskeletal system has been introduced by Taga et al. (1993). Locomotor control, for example, can be described in terms of sensorimotor integration as neural rhythm generators coordinate with the degrees of freedom of the musculoskeletal system. This meshes well with the embodied approach to cognitive science, which seeks a closer alignment between action, perception, and cognition.

Generally, the role of neural rhythm generator is given to central pattern generator (CPG) models. First proposed by Cohen et al. (1982) the CPG is an abstraction of the oscillatory pattern generators formed from neural circuits in the spinal cord of many animals and can generate patterns of activity without external input. The CPG model has been used to develop models of many forms of both rhythmic and non-rhythmic behavior (Collins and Richmond, 1994; Ijspeert, 2001; Fukuoka et al., 2004).

Importantly, these pattern generators are not just isolated homunculi and are capable of adaptation to changes in the environment via entrainment of their rhythmic patterns to other sources of activity. When this entrainment among components is extended to the entire brain-body-environment system, it is given the name “global entrainment” (Taga, 1994). This kind of interaction can be extended to arbitrary entrainment to non-linear processes in the “generalized synchrony” formulation (Friston and Frith, 2015).

Taga shows how bipedal locomotion can occur based on the dynamical patterns which arise from entrainment of a brain-body-environment system. The basic idea is that the CPG-generated rhythm affects the rhythmic activity of the limbs and is entrained by the sensory signals generated by their movement, resulting in a stable limit cycle in the state space of the coupled system. The intrinsic dynamics of the system are thus exploited to allow simple neural resources to “control” relatively complicated coordination of body parts, that is also robust and adaptive to perturbations. Here we see a solid foundation for building accounts of embodied cognition in terms of adaptive embodied/embedded behaviors.

As noted by Pitti et al. (2005), the emergence of any movement pattern thus would depend on specific forms of entrainment between neural and body/environment dynamics. The multiple sensorimotor loops ranging from simple monosynaptic reflex loops in the spinal cord to extended loops through the brain proper all must contribute to this in more complex animals, and it is then a question of how entrainment patterns in such a system lead to complex behavior.

In another model by Pitti et al. (2010), the model emulates the neuromodulator transmission from cortex to spinal cord that governs pattern generation in the CPG by matching the phase of neural controllers to body dynamics for either rhythmical or non-rhythmical gaits.

We highlight that there is vast potential to improve the complexity of modeled behaviors by studying these patterns in more detail. In particular, advancing from the simple limit cycle behaviors of spinal CPGs to more complex patterns in the full space of non-linear and chaotic dynamics is a must if whole-brain involving “higher cognition” is to be approached via this angle of research. To this end, we will later look at the use of such chaotic patterns to control embodied agents in more detail.

### Principle 2: Co-ordination of Coupled Components

In addition to entrainment of brain, body, and environment, internal components of the brain (modeled as neural oscillators in the studies we review here) must also be mutually entrained or coupled to one another in order for the system as a whole to enact complex dynamical behavior. We see a key example of this coupled chaotic field models (Kuniyoshi and Suzuki, 2004; Pitti et al., 2005, 2010; Kuniyoshi and Sangawa, 2006; Kinjo et al., 2008; Mori and Kuniyoshi, 2010), which connect chaotic elements together into a complex system of interacting components.

Here the problem then becomes one of coordination, as individual components must interact in the correct way to produce appropriate behavior generating patterns. This interplay takes the form of tendencies to either integrate with other components to fulfill collective behavior or segregate to act more independently in the context of any given task (Jirsa et al., 2010; Tognoli and Kelso, 2014). While we eschew the traditional notion of modular independent regions of the brain with fixed functionality, we can see how various components can take on dynamic roles in relation to other areas, allowing for the appropriate perception of and response to environmental states of affairs through self-organized dynamic behavior.

One example of segregation has been given by Rosslenbroich (2009), who points out that the locomotor neural processes of more evolved vertebrates are uncoupled from one another in order to act more independently, which is mirrored in Ren's model of adaptation to injury (Ren et al., 2015). In Ren's work, multiple CPGs act as redundant components that can synchronize with identical dynamics and act similarly to a single CPG, but during times of malfunction or disability (such as loss of use of a limb), the CPGs desynchronize and take on more independent roles to restabilize the behavior of the system (resulting in compensatory gaits of the agent).

Kuniyoshi's model supports varying degrees of coupling through a coupling parameter. When the coupling parameter ε is 0 (no coupling), each element behaves independently (chaotically), however, when coupling is enabled, dynamical structures can arise with various degrees of coherence or entrainment between elements, running the gamut between segregation and integration. Through modifying this parameter, it is possible to observe the metastable behavior of the system or different forms of entrainment between components. A similar phenomenon is observed in Shim and Husbands model (Shim and Husbands, 2012), whereby transient quiescence near pseudo-attractors of the state space (corresponding to forms of indirect coupling of neural elements through the environment) occurs in between intermittent states of chaoticity as the system searches for adaptive responses to perturbation. Kaluza's work shows how the dynamic cooperation of oscillator elements can act as memory by forming a “retrieval network” (Kaluza and Cioacǎ, 2012). Komarov treats the network of components as a network of “functional systems” which share responsibilities in agent control (Komarov et al., 2010).

It is important to note that such systems often exhibit more than just “desynchronized” or “synchronized” modes of behavior. In fact, in real organisms, much of the interesting behavior happens in the gradient between these two extremes (Santos et al., 2012). One particularly useful paradigm for discussing these issues is coordination dynamics, which considers varying levels of synchronization by using non-linear dynamical systems theory to describe the interaction between multiple oscillatory components and how that interaction changes with respect to key variables of the coupled system, such as the coupling strength and phase difference between components (Schöner and Kelso, 1988). Bressler and Kelso (2001) describe how coordination dynamics can be used to understand the relation between these interactions and cognitive task performance (see Bressler and Kelso, 2016 for a review). In Bressler and Kelso's view, the coordination dynamics of the brain thus relies on dynamically changing interdependencies of cortical areas, each of which has its own internal dynamics which contribute to the way in which that region interacts with other areas (Bressler, 1995). Thus, we can see how the dynamic formation and dissolution of large-scale collective units in the brain in “neurocognitive networks” (Meehan and Bressler, 2012) can correspond to particular behaviors as their dynamical patterns are entrained to those of the body and environmental situation, resulting in what would be described as “intelligent behavior.” Similar principles, as we have observed, also play a role in the adaptive behavior of minimally cognitive models of neurorobotics. How to extend current neurorobotic models with such neurocognitive networks remains an open question, but recent analysis of the relation between network properties and self-organizing dynamics by Park et al. (2017) shows promise in this direction.

### Principle 3: Discovering Adaptive Behaviors Through Chaotic Itinerancy

The brain-body-environment system, when considered as a coupled dynamical system, has an impossibly large state space. Only a subset of the state space, however, corresponds to successful adaptive behavior. It is a question then how to tractably lead an agent to these successful behavioral attractors. Crutchfield's idea of utilizing chaos as a means of generating variability comes into play here (Crutchfield, 2012), as itinerant trajectories through this state space can search for adaptive solutions.

Through itinerancy (Tsuda, 1991), the system sequentially wanders through a series of quasi-attractors and becomes entrained in each of them transiently (Kaneko and Tsuda, 2003). As we have discussed in **Principle 1**, when an unstable periodic orbit is found, chaos control can stabilize that orbit and use it as a new solution.

One idea has been to increase the level of chaos in the system to allow for chaotic itinerancy which can push the system to a new solution when it is stuck in maladaptive behavior. This is most prominent in Shim and Husbands' model, which uses “adaptive bifurcation” like an adjustable slider of chaos, increasing the degrees of freedom of the system to allow it to move along trajectories when it is necessary to move to a new state, but decreasing them (by reducing the chaoticity) when an adequate solution has been found. This is also seen in Steingrube et al. (2010), where the system is able to detect when it is trapped by an environmental obstacle and switch to chaotic dynamics autonomously, which then let the system follow rapid transients to more appropriate behaviors which can release the body from the obstacle. Further, in the model in Ren et al. (2015), we see how malfunction can lead to desynchronizing individual components, leading to transient chaotic behavior that eventually settles down into a new solution that is adaptive to the original perturbation. Finally, Park et al. (2017) explores chaotic itinerancy in the context of an embodied snake robot and investigates the coupled body-brain dynamic when several styles of oscillator networks (e.g., regular, small-world, scale-free, random) are employed, thus putting many of the principles outlined here (itinerancy, coupled dynamics, brain-body interaction) into practice.

### Principle 4: Storing Adaptive Solutions

Once adequate behavioral solutions have been found, they can be stored through the use of neural plasticity rules, so that when the same state is encountered again the agent can immediately switch to the correctly learned behavior. In Steingrube's work for example, in addition to the behavior generating CPGs, another learning neuron is modeled. This neuron can have varying levels of activity, each of which corresponds to a particular periodic orbit. Each orbit is then used as a particular gait in the body of the hexapod robot. By learning associations from sensory states to activity levels in this neuron, the model essentially forms a sensorimotor map. While this is a very primitive example of reactive behavior, it gives a solid foundation for developing more complicated sensorimotor contingencies on top of this framework.

In Shim and Husbands (2012), discovered patterns are memorized by wiring initially disconnected oscillators using ‘adaptive synchronization.” In Sussillo and Abbott (2009), so-called “FORCE learning” is used to modify synaptic strengths and stabilize chaotic spontaneous activity to desired activity patterns. In Kinjo et al. (2008), two aspects of learning are modeled: the compression of redundant motor commands and the mappings which couple controller, body, and environment. In Kuniyoshi and Sangawa (2006) models of sensory and motor cortex based on a dynamic variant of Kohonen self-organizing maps (Goodall et al., 1997; Chen, 1998) are employed, and associations are learned between sensory and motor areas and via cortical areas and “spinal” CPGs via Hebbian learning.

Taking the radical embodied view we would avoid treating the stored patterns seen here as “sensorimotor representations,” Instead, we should think of the formed maps as dynamical landscapes which allow for trajectories in cortical-spinal-body-environment space. In fact, there are such ways to analyze the dynamic landscape of motor cortex using dynamical systems theory and relate actions taken by the organism to the dynamic trajectory of activity in the neural population, leading to the consistent results outlined in Churchland et al. (2012). We will not delve into detail regarding this here but suggest that the dynamical systems analysis at the cortical scale is compatible with the full system analysis done in brain-body-environment models, and in fact may be enriched by taking these other factors into account.

Kuniyoshi's model, as well as other similar approaches such as by Yamada et al. (2016) which relate cortical dynamics to body dynamics, are hugely important examples of this style of analysis, and carry the potential for a parsimonious description of the “causal” chain of perception-action in terms of dynamical trajectories through the phase space of the entire system. The objective of learning then becomes the development of an effective dynamical landscape which reliably produces behavior that is adaptive to the agent's niche, as we describe next.

### Principle 5: Dependence on Environmental Information

Real organisms do not just make arbitrary movements, but actions aimed at particular goals. This behavior is adapted to a specific subset of the environment in which the organism operates, the so-called “niche” of the organism as outlined by Gibson (1979). We have considered the role of entrainment between the nervous system and the body, which reduces the search space of behavior by constraining it to those behaviors which are supported by the intrinsic dynamics of neural pattern generators and rhythmic limb movements. This approach also extends to the coupling with the environment, as we have seen for example in the previously mentioned models which utilize chaos to rapidly transition away from behaviors that do not coordinate well with the environment.

In the case of Steingrube's model, an energy based error term was introduced to let the agent know when to adapt its behavior to something more efficient. A similar feedback signal which evaluated the success of behavior was used in Shim and Husbands work to calibrate the level of chaoticity in the system. Kuniyoshi also briefly discusses how the internal coupling parameter ε could be coupled to external information, which in turn could specify the success of the current behavior in some way. More generally, we observe that in each case the agent is picking up some kind of “ecological information” (Gibson, 1979; Bruineberg et al., 2018) in order to modify its pattern of coordination with the environment.

So far the neurorobotics researcher has selected variables of interest to be optimized in order to design systems which can perceive and react to those aspects of the environment. But this bias presents an immediate limitation of these models. Clearly, the complexity of behavior by an agent would be limited by the kind of environmental pattern it was capable of perceiving or coupling its actions to. For example, there is a distinct advantage in the behavioral patterns mammals can take compared to amphibians and reptiles due to more precise limb placement based on visuomotor coordination with environmental features (Georgopoulos and Grillner, 1989).

Increased cortical interaction based on perceived specifying variables could allow for the more precise specification of the phases or frequencies of rhythmic movement at the spinal CPG level, and could thus be the starting point of increasingly “cognitive” behaviors such as reaching for and manipulating objects. This naturally leads to an account of perception in terms of possible actions and falls into the realm of affordance perception as outlined in ecological psychology. The perception by the agent of an environmental pattern would be built on its interactions with that pattern (O'Regan and Noë, 2001), as it senses important variables “through” its body dynamics (Iida and Pfeifer, 2006). This is in line with the prediction that the degree of coupling of the brain and body with environmental information in the form of entrainment or chaos control should be highly related to the interactions capable between agent and environment in practice (Dotov and Froese, 2018).

The aforementioned attention to certain information (specifying variables) has been discussed in terms of ecological psychology by Gibson, who calls the acquisition process of such variables “education of attention.” In the context of learning, Jacobs also points to the detection of non-optimalities in the brain-body-environment coupling through ecological information in his “Direct Learning” approach (Jacobs and Michaels, 2007), whereby relevant information is directly perceived in terms of how advantageous it is to the system's coupling, not unlike the models we have introduced earlier which detect measures of “error” or “performance.' In this scenario, making sense of the world is not just a process of perception-for-action but also a process of perception-for-learning, as coordination takes place over multiple timescales through the detection of, and adaptation to, non-optimalities.

What we would like to emphasize, however, is that this kind of coordination is not restricted to direct reflex-like behavior to immediate stimuli based on 1-to-1 sensorimotor maps. We propose that meaningful interaction with the world “as its own best model” (Brooks, 1991) can be enacted by the entire system over multiple timescales and that the key to achieving this is by better understanding the roles that controlled chaos and metastable morphing of entrainment patterns over time can play in directing agent behavior. Building on the principles we have outlined in this paper, we believe there is ample opportunity for expanding the state of the art in this direction.

## Conclusion and Remaining Issues

In this letter, we have outlined a series of principles employed by embodied neurorobotic models that rely on chaos control to find effective behavioral solutions. By explicitly outlining these principles we hope that their huge potential can be discovered by more researchers and that they can be extended beyond minimally cognitive models to more sophisticated demonstrations of embodied intelligence. What issues remain before we can make this transition?

For instance, we would like to build agents that can make finer discriminations of their environmental state based on constraints over multiple timescales, through an “education of attention” of relevant ecological information. One question that has not been fully explored is how the sense-making activities of an agent on the immediate timescale can lead to the biasing of its sensorimotor experiences, which ultimately lead to different developmental trajectories or specializations of skills. Thelen and Smith's dynamical systems view of behavioral learning (Thelen and Smith, 1994), as well as DiPaolo and Buhrmann's concept of trajectories through the sensorimotor space (Di Paolo et al., 2014), can be used to describe this process, but to this author's knowledge, a specific implementation of such an open-ended learner has not yet been attempted.

Another problem is that while attributing agents with greater amounts of autonomy is surely necessary for them to traverse the complexities of the real world, for practical applications they would need to achieve goals other than maintaining entrainment. It is an outstanding issue then how one could attribute agents with desirable goals while still allowing them to behave adaptively to their circumstances. One possible route to thinking about this is the extension of ecological psychology to cultural and social norms, whereby the direct perception of affordances in a “sociomaterial” world (van Dijk and Rietveld, 2017) can take place in the same non-representational fashion as the perception of physical ecological information. By imbuing agents with the ability to perceive regularities of a more abstract kind as opposed to just physical “laws,” we believe it may be possible for “skillful coping” in a social environment to take on the same character as the adaptive physical behavior outlined in this letter. In other words, the same chaos-based search, lock-in of well-performing patterns, rapid behavioral transitions, and perception in terms of contingencies or opportunities for action could apply to more abstract scenarios by conceiving of them in terms of perceiving and responding to pertinent ecological information.

While non-trivial, the above issues do not seem completely unsolvable by the style of models outlined in this letter. In fact they give us hope that a continuity may indeed be reached between reactive Brooksian models of “lower cognition” (Braitenberg, 1986; Brooks, 1991) and more complex social-rational agents capable of directly perceiving, and thus becoming entrained to, relevant aspects of the environment in terms of not just the physical but also the cultural. Here we posit that the key to achieving this is to surmount the countless degrees of freedom of the human behavioral space in an efficient way and that control over chaos may be a promising way to achieve this.

## Author Contributions

The author confirms being the sole contributor of this work and has approved it for publication.

### Conflict of Interest Statement

The author declares that the research was conducted in the absence of any commercial or financial relationships that could be construed as a potential conflict of interest.
